# RNA oxidation in chromatin modification and DNA-damage response following exposure to formaldehyde

**DOI:** 10.1038/s41598-020-73376-7

**Published:** 2020-10-06

**Authors:** Juan C. Gonzalez-Rivera, Mark W. Sherman, Dongyu S. Wang, Jamie C. L. Chuvalo-Abraham, Lea Hildebrandt Ruiz, Lydia M. Contreras

**Affiliations:** 1 McKetta Department of Chemical Engineering, University of Texas at Austin, Austin, TX 78714 USA; 2 Department of Cellular and Molecular Biology, University of Texas at Austin, Austin, TX 78714 USA

**Keywords:** RNA, High-throughput screening, Sequencing, Environmental impact, Biomarkers

## Abstract

Formaldehyde is an environmental and occupational chemical carcinogen implicated in the damage of proteins and nucleic acids. However, whether formaldehyde provokes modifications of RNAs such as 8-oxo-7,8-dihydroguanine (8-oxoG) and the role that these modifications play on conferring long-term adverse health effects remains unexplored. Here, we profile 8-oxoG modifications using RNA-immunoprecipitation and RNA sequencing (8-oxoG RIP-seq) to identify 343 RNA transcripts heavily enriched in oxidations in human bronchial epithelial BEAS-2B cell cultures exposed to 1 ppm formaldehyde for 2 h. RNA oxidation altered expression of many transcripts involved in chromatin modification and p53-mediated DNA-damage responses, two pathways that play key roles in sustaining genome integrity and typically deregulated in tumorigenesis. Given that these observations were identified in normal cells exhibiting minimal cell stress and death phenotypes (for example, lack of nuclear shrinkage, F-actin alterations or increased LDH activity); we hypothesize that oxidative modification of specific RNA transcripts following formaldehyde exposure denotes an early process occurring in carcinogenesis analogous to the oxidative events surfacing at early stages of neurodegenerative diseases. As such, we provide initial investigations of RNA oxidation as a potentially novel mechanism underlying formaldehyde-induced tumorigenesis.

## Introduction

Despite the potential hazard of exposure to individuals, formaldehyde, CH_2_O, is widely used in industrial and consumer products, including personal protective equipment (PPE) used by medical personnel^1–3^. Formaldehyde is a volatile organic compound (VOC) commonly used in the construction, clothing, automobile, and cosmetic industry and is often hailed for its properties in resins, flame retardants, and preservatives. While widespread in its uses and applications, formaldehyde has been linked to the occurrence of cancer by the U.S. Department of Labor’s Occupational Safety and Health Administration (OSHA) and by the International Agency for Research on Cancer^4,5^. OSHA guidelines indicate the total weight average (TWA) permissible exposure limit of 0.75 ppm over a 8-h total and the short term exposure limit as 2 ppm over a period of 15 min^5^. Outside of the United States, countries such as Norway, Germany, and Sweden have lower TWA thresholds (0.3–0.5 ppm)^6^. The consequences regarding exposure to formaldehyde are well-established; however, the investigation of causative mechanisms remains challenging because formaldehyde is quickly oxidized in the human body to formic acid, filtered by the kidneys, and excreted from the system^7^. Despite these challenges, several recent works, focused on the occupational exposure of formaldehyde in hair salons and tableware manufacturing facilities, have discovered DNA damage in blood comet assays even at low level exposures (< 0.3 ppm) to formaldehyde, which could result in potentially deleterious effects^8–10^. While studies like these demonstrate that low dose exposures may present risk at salons and factories, occupational exposure to formaldehyde in the garment, paper, and furniture manufacturing industries could reach up to 5.4 ppm, highlighting the importance of understanding how exposure correlates with disease^11^.

Multiple attempts have been made to detect and quantify levels of occupational exposure to formaldehyde and the impact it has on the human body; however, results are intrinsically unreliable due to variation in genetic background, exposures to other potentially confounding toxins, and generation of endogenous formaldehyde during processes such as cellular metabolism or cellular attachment^12,13^. Studies investigating the effects of formaldehyde exposure at the cellular level have been performed intensively, demonstrating radical damage to lipids, proteins, and DNA^14,15^. These studies highlight the formation of DNA-adducts and DNA–protein crosslinks in response to formaldehyde in association with carcinogenesis^16^; however, recent work has suggested that RNA also plays an important role in the cellular response to oxidative stress^17,18^. Reports investigating the reactivity to nucleic acids indicate that formaldehyde reacts with the amino groups of nucleobases and that the occlusion of nucleobase accessibility by secondary structure is a determining factor of reactivity^19,20^. Thus, the single stranded nature of RNA likely provides higher accessibility for attack by formaldehyde than double stranded DNA. Indeed, a recent study by Yamada et al*.* found that, when bound to the silane coupling reagent, bis[3‑(trimethoxysilyl)propyl]amine in vitro*,* RNA material accumulates formaldehyde over 3 times more efficiently than DNA material^21^.

The presence of RNA in the cellular and mitochondrial cytosol coupled with its association with metals makes it a vulnerable target for oxidation by reactive oxygen species (ROS)^22^. Since RNA is more readily oxidized than DNA, RNA oxidation could play a functional role in oxidative stress response^23–26^. Unlike the relatively prompt response mechanisms used by the cell to repair DNA oxidations by base excision repair pathways, oxidized RNA can remain in the cell for hours after a short insult of oxidative stress^17,27^. To this end, oxidative marks on RNA have been identified as an early indicator of cell death^17,23,28^. Damage to RNA induced by radicals could be detrimental to the cell, since RNA serves essential functions in protein synthesis, pathway regulation, and response to stress^29^. Compromise of these RNA functions could act as a modulating factor between oxidative stress and regulation of cellular pathways, potentially affecting the onset of disease.

Guanine is the most redox-sensitive RNA residue, resulting in the generation of 8-oxo-7,8-dihydroguanosine (8-oxoG) following exposure to ROS^30^; this represents one of the most common RNA alterations. Since all RNA transcripts consist of the same four nucleotides, it might be expected that RNA transcripts have similar vulnerability to oxidative stress based on their nucleotide composition, regardless of their template gene. However, recent studies have suggested that oxidation of RNA may be transcript-specific and independent of nucleotide composition, potentially impacting functionally relevant processes^23,28,31,32^. In addition to oxidative marks, recent work has shown that specific transcripts are differentially modified by methylations or other chemical groups and that these modifications occur at particular locations on transcripts^33–37^.

Increasingly sensitive methods to identify epitranscriptomics marks using chemical labels, biotinylation, and antibody pull-down such as MeRIP-seq, m^6^A-seq, CeU-seq, and other RNA modification-specific sequencing protocols have been developed and applied to find that the presence of modifications can be dynamically regulated by environmental stressors^18,34,36,38,39^. It is worth noting that particular transcripts have been characterized to have 8-oxoG enrichment relative to the total RNA pool, even under normal physiological conditions^40^. Consequently, the presence of 8-oxoG in RNA causes functional differences (i.e. in protein synthesis and aggregation) from its unoxidized counterpart^22,31^. As such, the oxidative enrichment of select transcripts under environmental stress, coupled with their prolonged half-life in the cell and the ensuing functional implications, could contribute to the initial deregulation of pathways critical for cellular function.

In this study, we exposed human bronchial epithelial BEAS-2B cells to air containing 1 ppm formaldehyde (representative of industrial levels) in an air–liquid interface (ALI) system. The human nasal epithelia is the main site of contact with gaseous formaldehyde and this cell type is commercially available as primary cells; however, because human nasal epithelium cells (HNEC) of standardized quality are not easy to get^41^, permanent model cell lines are more commonly used to assess the effect of formaldehyde exposure on respiratory cells^42–44^. Since bronchial cells demonstrate hyperactivity in epidemiological studies in response to formaldehyde exposure, BEAS-2B cell lines have been proposed as a biologically relevant cell type for exposure to highly-reactive pollutants such as ozone or formaldehyde^45^. The ALI system enables direct exposure of lung cells to tunable concentrations of purified chemicals, allowing precise measurement of biological response to controlled toxin exposure. The working concentration of 1 ppm formaldehyde represents a realistic, high exposure condition for occupational exposure^11^. Previous studies have used ALI exposures to study the effect of VOC mixtures^18,29,39,46^, cigarette smoke^47^, virus exposure^48^, and environmental pollutants^49^ on lung cells. Overall, our work identifies differentially oxidized transcripts utilizing 8-oxoG RNA-immunoprecipitation coupled with RNA sequencing (8-oxoG RIP-seq)^39^. Importantly, our analysis of transcripts enriched in oxidations suggests that functional pathways such as chromatin modification and DNA damage response may be particularly affected by formaldehyde-induced oxidative stress. Results yielding from these analyses could contribute to the development of novel RNA-based therapeutics aimed at reducing the impact of oxidations on the cell cycle and the progression of diseases. To the best of our knowledge, this is the first study that indicates RNA oxidations as components in the functional regulation of pathways that respond to cellular damage induced by formaldehyde exposures.

## Material and methods

### Culture of BEAS-2B cells

BEAS-2B cells were prepared for exposures according to protocols previously described by our lab^39,50^. Briefly, pre-coated T-75 culture flasks were used to initiate human bronchial epithelial BEAS-2B cells (ATCC CRL-9609) from cryopreserved cultures following manufacturer protocols. To measure cell density throughout this procedure, a Vi-Cell XR viability analyzer (Beckman Coulter, Brea, CA) was used to count cells from 0.6 ml of cell suspension. Approximately 225,000 cells were inoculated into 23 ml of complete Bronchial Epithelial Cell Growth Medium (BEGM, Lonza, Walkersville, MD, USD) and grown for 2 days at 37 °C in a humidified incubator containing 5% CO_2_. After 48 h, the medium was replaced with fresh BEGM and the cultures were incubated at 37 °C until they reached 70–80% confluence. Cell culture inserts (hydrophilic PTFE, 0.4 µm pore size, 30 mm diameter, EMD Millipore, Burlington, MA) were coated with 1 ml of 57 µg/ml of Bovine Collagen Type I (Advanced BioMatrix, Carlsbad, CA) in BEGM 24 h before seeding. The BEAS-2B cells were then passaged with a seeding density of 200,000 cells onto the inserts and then placed into 6-well cell culture plates (Corning Costar Clear Multiple Well Plates, Corning, NY) containing 1.1 ml BEGM. 0.8 ml BEGM were added to the apical side of the insert and the cultures were grown for 24 h at 37 °C in a humidified incubator. Two hours before exposure, the media contained in the well was replaced with fresh BEGM media and the media on the apical side of the insert was removed completely to prepare the cultures for the air–liquid interface chambers.

### Air–liquid interface (ALI) exposures of BEAS-2B cells

Two polycarbonate modular cell exposure chambers (MIC-101 Billups-Rothenberg, San Diego, CA) were prepared to house treatment and control samples for exposure experiments, similar to exposure experiments previously performed by our lab^18,39^. Prior to each exposure, the chambers were flushed with 0.15–0.35%v O_3_ for 15–20 min at ambient temperature and humidity at a flow rate of 2 L/min to reduce contamination by plasticizer residues, left overnight, and flushed with clean air for 20 min to displace residual O_3_.

Formaldehyde gas was generated via thermal decomposition of paraformaldehyde powder (Alfa Aesar, 97%) similar to previously published methods for formaldehyde generation^44^. Briefly, paraformaldehyde powder was measured using an analytical balance (ALF 64, Fisher Scientific) to reach an approximate gas concentration of 1 ppm (complete details in Supplemental Methods). Paraformaldehyde was placed inside the head plug of a 316 stainless steel Swagelok tee, wrapped in heating tape (Omega Engineering, HTWC101-010), and injected at > 40% output with a flow rate of 2 L/min with ultra-high purity (UHP) N_2_ through the shoulders of the tee into an environmental chamber (Figure S1). A 360° bend in tubing was placed immediately downstream of the injection tee to obstruct stray particles. Clean air was generated using an Advanced Apparatus Development Company (AADCO) instruments’ high purity air generator. Formaldehyde was mixed with humidified clean air inside the environmental chamber to reach the targeted gas-phase concentrations. A mix of 0.08 L/min CO_2_ and 1.52 L/min formaldehyde-containing air was pumped through the formaldehyde environmental reaction chamber for a total flow rate of 2 L/min. In parallel, a mix of 0.08 L/min CO_2_ and 1.52 L/min humidified clean air was pumped through the clean air exposure chamber. Gas phase compounds (formaldehyde, methanol, ethanol, acetaldehyde, formic acid, glycolic acid, lactic acid) were monitored throughout the experiment by chemical ionization mass spectrometry (CIMS, Aerodyne, Billerica, MA, USA).

Cells were placed in the chamber, sealed, and exposed to either formaldehyde air or clean air pulled from the environmental chamber for two hours. Media was then replaced, and cells were allowed to recover for 6 h at 37 °C in a humidified incubator under an atmosphere containing 5% CO_2_ until RNA was extracted.

### Cytotoxicity assay

After six hours of recovery from exposure in fresh BGEM media, the basolateral medium from each well was collected and frozen at − 80 °C until the day of analysis. Cellular membrane damage was measured by detection of lactase dehydrogenase (LDH) in the cellular medium using a colorimetric assay (LDH Cytotoxicity Detection Kit, Takara Bio, Japan). LDH is an enzyme released into media after plasma membrane damage and is proposed to increase proportionally to the number of dead cells^51^. Absorbance of the assay was measured at 491 nm for 30 min at 25 °C using a Cytation3 plate reader (Biotek, Winooski, VT).

### Confocal microscopy

Confocal microscopy images of BEAS-2B cells adhered to hydrophilic PTFE membranes were acquired as previously described^39^. Prior to treatment of the cells, the insert’s membrane was cut off from the plastic insert by making an incision around the edge of the membrane. Each membrane was then placed onto a microscope slide mounted in a petri dish with cells facing upward. Cells were fixed in 1 ml of 3.7% formaldehyde solution in phosphate buffer solution pH 7.4 (PBS, Thermo Fisher Scientific, Waltham, MA) for 15 min at 37 °C. After fixation, the formaldehyde solution was discarded, and the membrane was washed three times with 1 ml of PBS pre-warmed to 37 °C. Then, 1 ml of 0.1% Triton-X-100 (Sigma) in PBS was placed onto the membrane for 4 min and washed with 1 ml PBS three times. The membrane was then pre-incubated with 1 ml of 1% bovine serum albumin (BSA) in PBS for 20 min, prior to adding the phallotoxin staining solution. To stain F-actin in the cells, 10 µl of Alexa Fluor 594 Phalloidin solution (Thermo Fisher Scientific, Waltham, MA) was diluted into 400 µL of PBS with 1% BSA solution. The staining solution was placed onto the membrane for 20 min at room temperature and protected from light to prevent photobleaching. The fluorescent media was aspirated and washed three times with PBS. Once each membrane was stained, a drop of ProLong Gold Antifade Mountant with DAPI (Thermo Fisher Scientific, Waltham, MA) was placed onto the membrane. A coverslip was positioned on top of the membrane, and then the edges of each coverslip were sealed with clear nail polish and left to dry. Specimens were stored in the dark at 4 °C until the day of analysis. Confocal microscopy for analysis was performed using a Zeiss LSM 710 Confocal Microscope. Five or more images were acquired in random locations and captured using Zen Pro software with a 63 × oil objective and filters for DAPI and Alexa 594.

### Image analysis

The cytosolic area was estimated from the phalloidin Alexa 594-stained F-actin surface and the nucleic area from the DAPI-stained DNA. The area was quantified in Fiji/ImageJ by drawing the outline of the cell with the free hand pencil tool in at least 5 cells in 3 confocal images (63 × magnification) selected for each biological replicate and condition. The F-actin organization around the nucleus and plasma membrane was quantified using Fibriltool plugin in Fiji according to the described protocol^52^. This analysis was conducted in 3 confocal images (63 × magnification) selected for each biological replicate and condition. The anisotropic score was computed on 5 or more cells per image by drawing an area of interest of approximately 5 µm by 10 µm.

### RNA preparation

Following exposure, the apical side of each membrane was treated with 1 ml of TRIzol (Invitrogen, Carlsbad, CA) and gently mixed to ensure thorough lysis of cell culture. Lysate was collected and frozen until the day of the extraction. TRIzol RNA extraction was conducted following manufacturer instructions with freshly prepared ethanol (200 Proof, OmniPur, EMD Millipore, Burlington, MA), isopropanol (molecular biology grade, IBI Scientific, Dubuque, IA) and nuclease-free water (Ambion, Austin, TX) purged of oxygen with ultra-high purity N_2_. Briefly, TRIzol aliquots were thawed on ice and 1 ml of chloroform (HPLC grade, J.T.Baker, Phillipsburg, NJ) was added to induce phase separation. Soluble RNA in the aqueous phase was precipitated in 0.5 ml isopropanol overnight at − 20 °C with 1.6 µl glycogen (GlycoBlue, Thermo Fisher Scientific, Waltham, MA) as a carrier. Following precipitation, the pellet was washed twice with 1 ml 95% ethanol and air-dried. The purified RNA was incubated with DNAse I (New England Biolabs, Ipswich, MA) following the manufacturer’s protocol. RNA was then re-extracted with 200 µl of 25:24:1 mixture of phenol/chloroform/isoamyl alcohol (Fisher BioReagents, Hampton, NH) followed by a chloroform extraction and an isopropanol precipitation as described above. After DNase I treatment of RNA, ribosomal RNA (rRNA) was depleted using Ribo-Zero Gold rRNA Removal Kit (Illumina, San Diego, CA) as described by the manufacturer’s protocol to produce the formaldehyde air and clean air input RNA samples (FA_IN_ and CA_IN_). Depletion of rRNA was validated by Agilent 2100 Bioanalyzer (Agilent, Santa Clara, CA) and all samples surpassed an RNA Integrity Number (RIN) threshold of 7.00. This protocol has been adapted from other published work by our group^53–55^.

Immunoprecipitations of 8-oxoG-containing RNA transcripts were performed in biological duplicates for clean air (CA) and formaldehyde (FA) conditions, following a protocol previously established in our group^18,39^. All buffers were prepared fresh from concentrated stocks on the day of pulldown experiments and purged of O_2_ as described above. A portion of the input RNA was incubated with 12.5 µg of 8-oxo-7,8-dihydroguanosine (8-oxoG) monoclonal antibody (0.5 mg/ml, clone 15A3, Trevigen, Gaithersburg, MD) in immunoprecipitation (IP) buffer (10 mM Tris pH 7.4, 150 mM NaCl, 0.1% IGEPAL, and 200 units/ml SUPERaseIn RNA inhibitor [Invitrogen, Carlsbad, CA]) in a 1 ml reaction volume for 2 h at 4 °C with rotation. The 8-oxoG antibody binds specifically to 8-oxoG-containing transcripts directly without mediation through a RNA-binding protein^56^. SureBeads Protein A magnetic beads (Biorad, Hercules, CA) were washed according to manufacturer’s protocol and blocked in IP buffer supplemented with 0.5 mg/ml bovine serum albumin (BSA) for two hours at room temperature. After washing beads twice in IP buffer, the beads were resuspended in IP buffer, mixed with the RNA-antibody reaction and incubated for 2 h at 4 °C with rotation. Next, the beads were washed three more times in IP buffer before two rounds of competitive elution were performed. Each elution consisted of incubation of the beads with 108 µg of 8-oxo-dG (Cayman Chemical, Ann Arbor, MI) in IP buffer for 1 h at 4 °C with rotation. The elution volume was then cleaned up using the RNA Clean and Concentrator-5 kit (Zymo Research, Irvine, CA) to isolate Clean Air-immunoprecipitated oxidized RNA (CA_IP_) and Formaldehyde-immunoprecipitated oxidized RNA (FA_IP_).

### RNA sequencing

Libraries for CA_Input_, FA_Input_, CA_IP_, and FA_IP_ were prepared using the NEBNext Small RNA kit (NEB, Ipswich, MA) by the Genomic Sequencing and Analysis Facility (GSAF) at the University of Texas at Austin. Sequencing was performed on an Illumina HiSeq 4000 to yield 75-bp reads with an average read depth of 31 M reads for pull-downs and 56 M reads for total RNA samples. Raw sequencing results are available in the NCBI GEO database (Accession number: GSE148377).

### Data analysis

Raw sequencing data was acquired from the GSAF and high run quality was visually confirmed using FastQC (https://www.bioinformatics.babraham.ac.uk/index.html). Runs were processed with Cutadapt to remove primer and adaptor sequences^57,58^. After trimming, reads were re-assessed with FastQC for read quality and the removal of repetitive sequences was confirmed. Trimmed reads were then aligned with Spliced Transcripts Alignment to a Reference (STAR) aligner^59^. STAR was chosen over other mapping programs such as Tophat2, HISAT, bwa, and bowtie for its ability to identify novel transcript isoforms via a two-pass mapping approach.

The full description and code used to analyze transcriptomic data is described in Supplemental Methods. Briefly, a STAR genome index was constructed utilizing the ENSEBL GRCh38.p12 primary genome assembly (ftp://ftp.ensembl.org/pub/release-94/fasta/homo_sapiens/dna/Homo_sapiens.GRCh38.dna_sm.primary_assembly.fa.gz) with the corresponding annotations (ftp://ftp.ensembl.org/pub/release-94/gtf/homo_sapiens/Homo_sapiens.GRCh38.94.gtf.gz). The genome index was used as a reference for first pass mapping of the trimmed reads to identify and annotate novel splice junctions. The novel splice junction database was then used in conjunction with the genome index for second pass mapping of trimmed reads to create an Aligned.to.Transcriptome.bam output file. Read alignments were visually inspected for proper alignment of transcripts to annotated genes by Integrative Genomics Viewer and the number of reads collected for each splice variant was calculated using RSEM^60–62^. RSEM was chosen for read counting because it uses the SAM/BAM Aligned.toTranscriptome output file from the STAR aligner as input to account for novel isoforms generated during the two-pass mapping approach. The RSEM reference file was prepared using ENSEBL GRCh38.p12 and its corresponding annotation described above to calculate expression of each splice variant from the STAR output bam file.

### Annotation and functional analysis

The tximport package was used to import the RSEM results file into R, allowing assessment of each transcript generated by STAR^63,64^. Statistical analysis of differential expression and 8-oxoG enrichment was performed with DESeq2 in R version 3.5 using modified steps in the DESeq2 manual and help page^65^. DESeq2 utilizes the transcript abundance across different conditions to calculate the statistical significance of transcript expression level changes. FA_IN_ and CA_IN_ were compared for standard differential expression analysis using changes in transcript levels in response to formaldehyde exposure. Transcripts were identified as differentially expressed (comparing FA_IN_ and CA_IN_) if their p_adj_ < 0.05 and their log_2_FC >|2|. DESeq2′s p_adj_ was used for determining statistical significance because it utilizes the Benjamini–Hochberg method to control for type I error due to multiple comparisons. A p_adj_ cutoff of less than 0.05 and a fold change greater than 4 (log_2_FC >|2|) was chosen so that only relevant genes were included the downstream functional network analyses.

Comparisons of log_2_(fold change) values between FA_IP_ and FA_IN_ (referred to as FA_FC_) as well as CA_IP_ and CA_IN_ (referred to as CA_FC_) were calculated to identify transcripts that were differentially oxidized relative to their input RNA. By normalizing each oxidized transcript isolated by immunoprecipitation relative to the expression of its corresponding transcript abundance in the input pool, relative enrichment of oxidation for individual transcripts could be calculated^66^. Biological replicates reduced noise due to nonspecific binding of antibodies to transcripts and minimized bias within sequencing reactions^67^. The use of proportional enrichment of transcripts in formaldehyde exposures relative to clean air controls helped to discriminate formaldehyde-induced oxidations from background oxidations^66^. For this reason, a comparison between CA_IP_ and FA_IP_ was not performed because IP requires input RNA as a frame of reference for enrichment of individual transcripts relative to expression of the transcript from the input RNA pool.

To identify transcripts enriched in oxidation resulting from oxidative stress associated with the formaldehyde exposure, candidate transcripts (p_adj_ < 0.05) resulting from the DESeq2 analysis between immunoprecipitated and input RNA pools were filtered for further analysis. Differences in log_2_ fold changes between these transcripts in the formaldehyde treatment and their corresponding transcripts in the clean air condition were calculated by subtracting the DESeq2-generated log_2_FC value of clean air controls from that of formaldehyde exposed samples for each transcript (∆log_2_FC = FA_log2FC_ − CA_log2FC_), similar to that performed by Soetanto et al.^68^. Log_2_FC values of 0.00 were raised to 0.01 to enable log calculations without influencing count data. The difference between FA_log2FC_ and CA_log2FC_ was used to calculate the relative magnitude of oxidation for each transcript between clean air and formaldehyde air exposures. A cutoff value of ∆log_2_FC > |2| was used to identify differentially oxidized transcripts associated with formaldehyde exposure and were used for further functional analyses.

Raw counts from RSEM were manually inspected to ensure that the major drivers of differentiation were not due to noise from variation in low transcript counts (minimum estimated counts above 0 were 22.99 and 16.32 for differential expression and oxidation enrichment analyses, respectively). Protein name information for each transcript was retrieved using the BiomaRt R package with the Ensembl market database setting^69^. Transcripts identified as differentially expressed or enriched in oxidations were then used for downstream analyses of protein interactions, cellular/biological gene ontology, and functional pathways.

Strong clustering of network associations have been used to infer functional relationships amongst proteins, as groups of strongly interacting genes can be indicative of ongoing cellular processes^70,71^. To elucidate potential functional interactions among transcripts identified by the differential expression and oxidative enrichment analyses, STRING database was used to identify known interactions amongst transcripts identified by the ∆log_2_FC filtering steps outlined above^72,73^. Protein–protein interaction (PPI) networks were constructed in Cytoscape StringApp^74,75^. Furthermore, STRING enrichment within Cytoscape was used to assess association with potential functional roles of transcripts in response to formaldehyde exposure. Databases with relevant information for gene ontology (GO Biological Processes 2018, GO Molecular Function 2018, GO Cellular Component 2018) and molecular pathways (Reactome 2016) were compiled and filtered to remove redundant terms and to determine statistical significance of association with the genes assessed (p_adj_ < 0.05). The pathways and GO terms identified were further investigated through literature review for relatedness and experimental relevance.

### Statistics and reproducibility

We conducted all described measurements at least twice using independent and biological replicates. The experiments were not randomized, and the researchers were not blinded during experiments. All data was presented as the mean ± one standard deviation. Statistical analysis between groups was performed in Rstudio software (V 1.2.5042) using the function stat_compare_means and determined by t-test (two-tail homoscedastic).

## Results

### Minimal phenotypic changes in BEAS-2B cell line exposed to 1 ppm formaldehyde exposure

We exposed biological triplicates of human bronchial epithelial BEAS-2B cell cultures to formaldehyde following a previously described experimental design^18,39^. Cells were grown in collagen-coated inserts with a seeding density of 200,000 cells and incubated for 24 h to reach 70–80% confluence. Two hours before exposure, the medium from the apical cell surface was fully removed and the medium from the basolateral surface was renewed with fresh complete medium. Cells were then put into an exposure chamber and subjected to humidified clean air (control condition) or humidified air containing 1 ppm formaldehyde for 2 h at 2 L/min. An air–liquid interface (ALI) system was used to allow direct exposure of the lung cells to the desired concentration of toxin (Figure S1). For this study, we chose 1 ppm formaldehyde exposures over the course of 2 h to reflect occupational exposure conditions from individuals in the garment (0.9–2.7 ppm), furniture (0.4–5.4 ppm), and utensil (0.5–2.6 ppm) manufacturing industries^11,76^. Following exposure, cells were allowed to recover with fresh media for 6 h.

**Figure 1:**
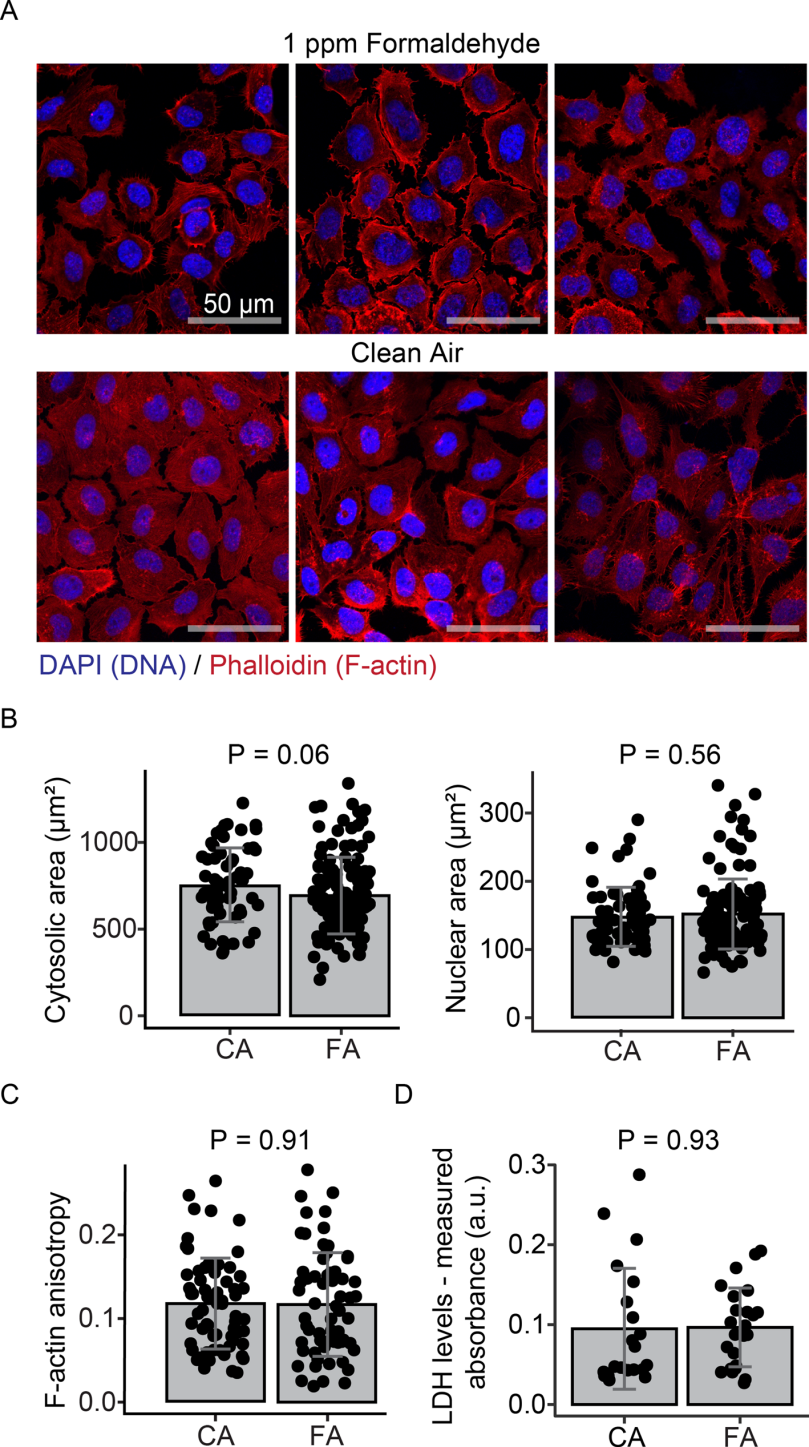
Minimal toxicity effects in BEAS-2B cells exposed to 1 ppm formaldehyde. (**A**) Representative images of BEAS-2B cells treated with formaldehyde and clean air (N = 3). Confocal fluorescent microscopy of cells stained with Alexa Fluor 594 phalloidin for F-actin (red) and DAPI for nuclei (blue) using an objective magnification of 63X. (**B**) No significant changes in cytosol and nuclear size were found after formaldehyde (FA) treatment as compared to clean air (CA) controls (N > 65 cells). (**C**) No significant changes in F-actin organization in exposed cells. An anisotropy score of 0 is given for no order (purely isotropic fibrils), and 1 is given for perfectly parallel fibrils (purely anisotropic arrays). This analysis was conducted in 10 µm × 5 µm regions on 65 cells per treatment. (**D**) LDH activity levels assayed after two hours of exposure and six hours recovery show no significant differences in exposed cells. Statistical difference was computed by *t*-test analysis and error bars are expressed as one standard deviation.

We examined morphological features indicative of cellular stress such as nuclear shrinking^77^ and alterations in actin filaments^78^ using confocal microscopy (Fig. 1A). We analyzed the cytosol and nuclear area of exposed cells demonstrating no significant alterations in size relative to control samples (Fig. 1B). We further quantified the heterogeneity of F-actin orientations (or F-actin anisotropy) in cortical actin filaments using FibrilTool^52^. As seen in Fig. 1C, our results show no significant alterations to cellular cytoskeletal structures. Moreover, we analyzed cytotoxicity effects of formaldehyde exposure in BEAS-2B cells with lactase dehydrogenase (LDH) activity assays (Fig. 1D). We observed no significant differences in LDH activity in the cellular media relative to control samples. Taken together, these data illustrate that minimal phenotypic alterations were observed in cells treated with 1 ppm formaldehyde.

### Profiling 8-oxoG modifications shows oxidation of chromatin modification and DNA-damage response transcripts following 1 ppm formaldehyde exposure

**Figure 2:**
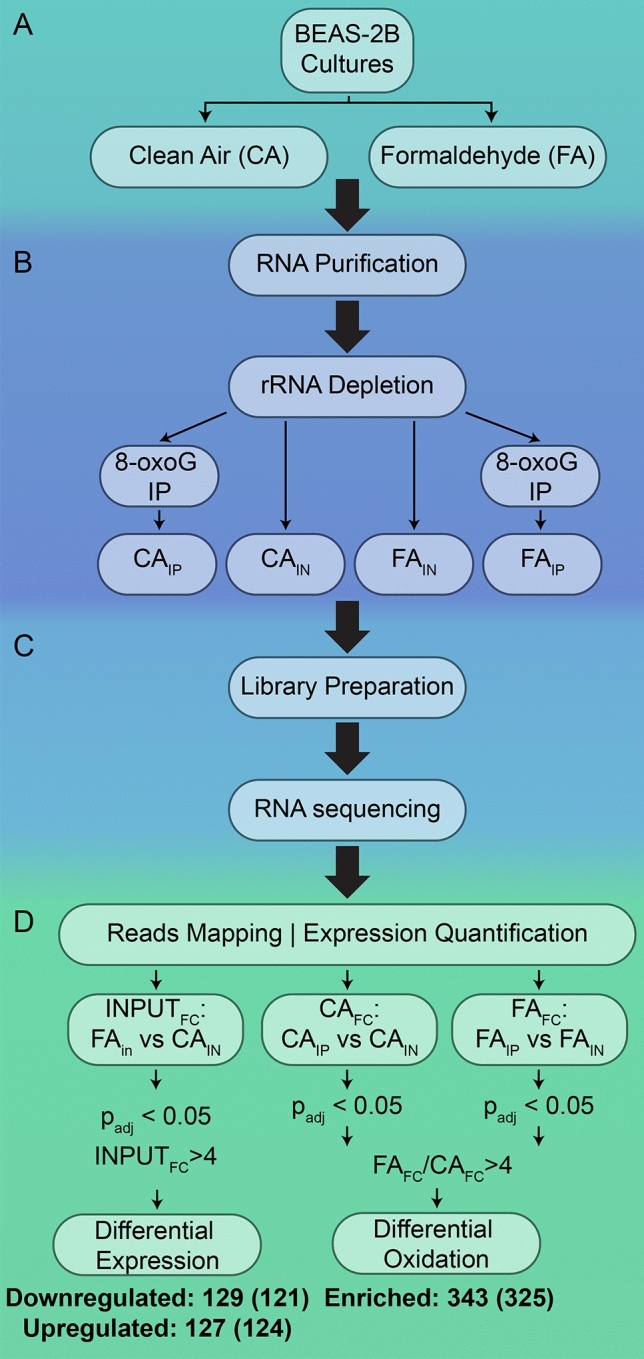
Schematic of 8-oxoG RIP-seq experimental workflow. (**A**) BEAS-2B cell cultures were exposed to 1 ppm formaldehyde or clean air. (**B**) Total RNA was extracted and ribosomal RNA (rRNA) was selectively depleted to yield a pool of enriched whole transcriptome RNA. A fraction of this pool was mixed with an anti-8-oxo-7,8-dihydroguanosine (8-oxoG) antibody followed by protein A magnetic beads. The antibody-bound RNA was recovered by competitive elution with excess of free 8-oxoG nucleotides. (**C**) Both pools, the transcriptome pool and the 8-oxoG transcript pool from each condition, were submitted for Illumina RNA sequencing. (**D**) Transcript sequences were processed and statistically identified. The number of transcripts identified in the analyses are described in the box of the test. Numbers in parentheses indicate the number of unique gene products identified by Enrichr corresponding to the transcript set.

We profiled 8-oxoG-modified transcripts using an RNA-sequencing approach coupled with 8-oxoG immunoprecipitation that was recently established by our lab^18,39^. Two biological replicates were used for clean air (CA) and 1 ppm formaldehyde (FA) exposures (Fig. 2A). After RNA extraction and ribosomal RNA (rRNA) depletion, the resulting RNA pool for each treatment was then split into two fractions. One fraction for each treatment—referred in Fig. 2B as CA_IN_ and FA_IN_– was used to quantify total RNA abundance of transcripts and served as a reference to measure relative enrichment following immunoprecipitation. The second fraction for each treatment—CA_IP_ and FA_IP_, respectively – was used to isolate 8-oxoG-containing transcripts via immunoprecipitation with an anti-8-oxoG antibody (clone 15A3). The clone 15A3 can recognize 8-oxoG in DNA and RNA^28,79–81^, and it has been applied and validated for 8-oxoG immunoprecipitation of miRNA and mRNAs^28,81^. These samples were used for library preparation and sequencing (Fig. 2C) and the reads were processed and then analyzed with DESeq2^82^. To assess transcript enrichment, we conducted pairwise comparisons, e.g., CA_IP_ with CA_IN_, FA_IP_ with FA_IN_, and FA_IN_ with CA_IN_ (Fig. 2D). Full experimental details are described in the material and methods section.

To identify 8-oxoG transcripts that were significantly oxidized in the formaldehyde exposure, we first applied a threshold of p_adj_ < 0.05 enrichment compared to input in at least one condition (i.e., CA_IP_ with CA_IN_ or FA_IP_ with FA_IN_). Subsequently, we applied a cutoff of fold-change (FC) difference between the resulting FA pool (FA_FC_) and CA pool (CA_FC_) greater than 4 (i.e., FA_FC_/CA_FC_ > 4, see Fig. 2D). By applying these thresholds, we removed any potential unintended drivers of differentiation (e.g., non-specific interactions, antibody cross-reactivity and artifactual 8-oxoG formation during library preparation)^18,39^. Nine transcripts detected in the FA pool but not in the CA pool were also included, since detection in the formaldehyde treatment alone demonstrated enrichment. This analysis identified 343 transcripts as enriched in oxidation from formaldehyde-exposed cultures relative to the same transcripts from clean air samples (full list of transcripts listed in Data S1). Over 70% of the 343 oxidation enriched transcripts were protein-encoding, and ten genes had more than one transcript isoform in the oxidation enriched set (e.g. ACTB, BRCA1, DDX3X, FN1, LPP, MLLT10, SMARCA4, SNORD3B-2, SNU13 and ZBTB14).

Principal component analysis of 8-oxoG RIP-seq data (i.e., CA_IP_ with CA_IN_ and FA_IP_ with FA_IN_) showed that the major drivers of variance were the 8-oxoG immunoprecipitation procedure and the treatment condition (Figure S2A and S2B). Moreover, the volcano plots show unbiased distribution of immunoprecipitated transcripts (Figure S2C and S2D) consistent with previous 8-oxoG RIP-seq datasets^39^, further validating the effective execution of our 8-oxoG RIP-seq experiments.

**Figure 3:**
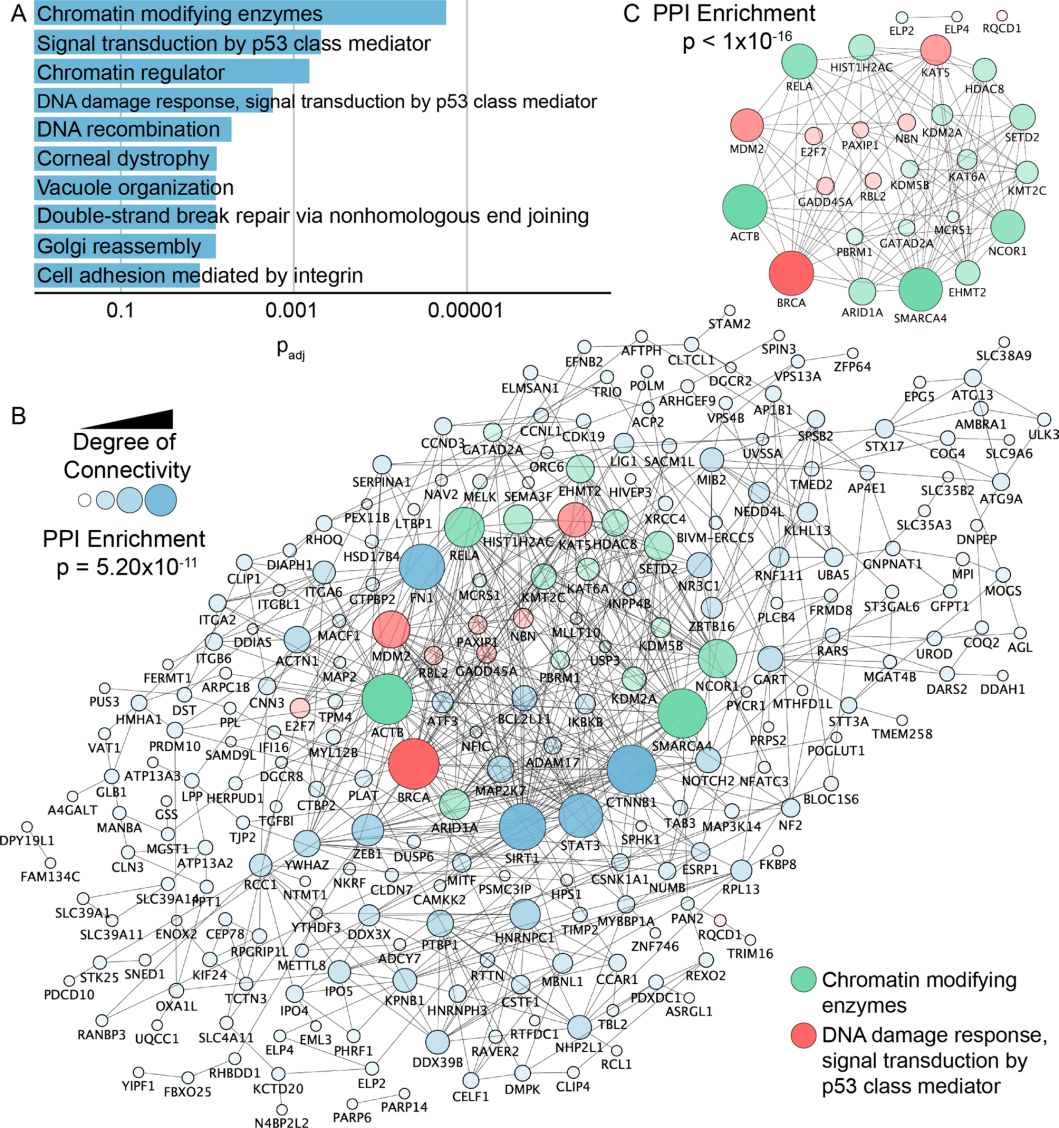
Functional analysis of 8-oxoG enriched transcripts shows RNA oxidation in chromatin regulation and DNA-damage repair transcripts. (**A**) Functional pathway analysis and GO associated terms identified by StringApp in Cytoscape based on transcripts enriched in oxidation following formaldehyde exposure of BEAS-2B cells (p_adj_ < 0.05). Redundant pathways were removed for clarity. Individual transcripts associated with each pathway are described in Data S2. (**B**) STRING-DB analysis of gene products from oxidation enriched transcripts shows significantly more interactions than expected due to chance (p = 5.20 × 10^–11^) following exposure to 1 ppm formaldehyde. (**C**) Gene products from the oxidation enriched set that belong to the chromatin modifying enzymes (green shade), and to the DNA damage response, signal transduction by p53 class mediator (red shade) share a high degree of connectivity in the protein–protein interaction network (p < 1 × 10^–16^). Larger node size and darker fill shade indicate higher connectivity.

The identification of transcript enrichment in 8-oxoG immunoprecipitated fractions relative to the total RNA pool enables novel insight about cellular processes that may be potentially impacted by nucleotide modifications imposed on existing RNA transcripts. We performed pathway and gene ontology (GO) enrichment analyses using the StringApp in Cytoscape^72,74,75^. Significant associations below the cutoff (p_adj_ < 0.05) were ranked by their p_adj_ to provide context of relevant gene ontologies for terms potentially impacted by RNA oxidation (Fig. 3A). The patterns do not appear to be driven by enrichment of single genes associated with multiple terms and pathways; rather, diverse suites of transcripts contribute to enrichment of terms that include few general members. Specifically, the resulting functional assessment and GO analysis indicates oxidative enrichment of transcripts associated with chromatin regulation, cell signaling, cell adhesion and DNA processes such as strand break repair and DNA recombination. Full details of significant GO terms and pathways are provided in Data S2.

To explore relationships between transcripts identified as enriched in oxidations from the formaldehyde treated cells relative to the clean air controls, we performed a network analysis in Cytoscape^75^. Importantly, this analysis identified significantly more interactions than expected due to chance (p = 5.20 × 10^–11^, Fig. 3B), suggesting that functional cellular processes could be differentially affected by transcript oxidation^71,72^. Closer inspection of the most densely connected nodes highlight transcripts such as Actin, cytoplasmic 1 (ACTB) with 31 connections, Breast cancer type 1 susceptibility protein (BRCA1) with 31 connections, Catenin beta 1 (CTNNB1) with 30 connections, Transcription activator BRG1 (SMARCA4) with 30 connections, and NAD-dependent protein deacetylase sirtuin-1 (SIRT1) with 28 connections (Data S3). These transcripts are mainly associated with chromatin modifying enzymes and DNA damage response pathways. Importantly, the subset of oxidation enriched transcripts that constitute these pathways share stronger interconnectivity than expected due to chance (p < 1 × 10^–16^) (Fig. 3C).

Taken together, our data provides evidence of oxidative modification in 343 transcripts (out of > 140,000 total transcripts) in BEAS-2B cells after formaldehyde exposure. Importantly, we show that multiple functional associations affected by oxidations become apparent in relatively few functional categories, supporting recent work that proposes that RNA oxidation is selective, rather than randomly distributed across the transcriptome^28,34,83^. Specifically, we demonstrate that formaldehyde induces RNA oxidation in cellular processes specific to the role of chromatin regulation and DNA-damage response. Oxidative marks and the resulting defects in transcripts associated with these pathways may make them early markers for cell function and fate, as previously suggested by Shan *et al*^31^.

### 8-oxoG-modified transcripts in the chromatin regulation and DNA-damage response by p53 pathways are differentially downregulated

Our assessment of differential expression between input RNA samples (CA_IN_ and FA_IN_), applying p_adj_ < 0.05 and log_2_FC > |2| cutoffs, identified 256 differentially expressed transcripts from cultures exposed to formaldehyde relative to clean air samples (Figure S3). Of these differentially expressed transcripts, 127 were upregulated (Data S4) and 129 were downregulated (Data S5). Oxidation and differential expression are considered to be mutually exclusive from each other because oxidative stress by formaldehyde is presumed to impact the suite of transcripts present in the cell at the time of insult, regardless of their regulation following the event. Interestingly, 52 of the 343 transcripts from the oxidative enrichment analysis were identified in the differential expression analysis as downregulated (Data S6); none of the 343 oxidative enriched transcripts were identified as significantly upregulated. Of these 52 downregulated and oxidation enriched transcripts, 13 are involved in the chromatin modifying enzymes pathway and four are involved in DNA damage response, signaling transduction by p53 class mediator (Table 1). One transcript coding for Histone acetyltransferase KAT5 (KAT5) is present in both pathways. Moreover, we found that among the highly connected gene products, two oxidation enriched transcripts of ACTB and BRCA1 and one oxidation enriched transcript of SMARCA4 (out of two oxidation enriched transcripts for this gene product) were differentially downregulated. It is important to note that replacement of oxidized transcripts by the production of non-oxidized transcripts in response to formaldehyde exposure (upregulation) would likely supersede the signal from oxidized transcripts generated from the stress event. Downregulated transcripts likely appear to harbor more extensive oxidation because they are not readily replaced and potentially impact biological function, since the results show significant oxidative enrichment of transcripts that code for proteins associated with oxidative stress response pathways.

**Table 1:** Fold-change in 8-oxoG enrichment and differential expression (DE) in the chromatin modification and DNA-damage response pathways after formaldehyde exposure.

Pathway	HGNC symbol	Ensembl transcript ID	8-oxoG enrichment log_2_(FC)	DE log_2_(FC)	DE p_adj_*
Chromatin modifying enzymes	HIST1H2AC	ENST00000314088	18.6	9.16	**0.0221**
	ELP4	ENST00000638347	8.28	7.77	**< 0.0001**
	HDAC8	ENST00000647980	7.28	7.61	**0.0001**
	MCRS1	ENST00000551625	6.81	6.99	**0.0012**
	KAT5	ENST00000534650	4.44	6.67	**0.0048**
	EHMT2	ENST00000395728	6.30	6.37	**0.0127**
	ACTB	ENST00000473257	11.3	6.14	**0.0214**
	SMARCA4	ENST00000642726	8.77	5.98	**0.0018**
	ACTB	ENST00000425660	8.69	5.48	**0.0732**
	KDM2A	ENST00000526258	5.06	5.06	0.2846
	ARID1A	ENST00000457599	15.4	5.01	**0.0948**
	KDM5B	ENST00000648056	9.84	4.24	0.1592
	SETD2	ENST00000445387	3.15	3.96	**0.0085**
	KMT2C	ENST00000355193	6.66	3.18	**< 0.0001**
	KAT6A	ENST00000648030	6.09	3.10	0.5063
	PBRM1	ENST00000410007	9.90	2.89	0.5539
	SMARCA4	ENST00000646484	8.53	1.53	**0.0229**
	RELA	ENST00000612991	8.38	1.52	**0.0235**
	ELP2	ENST00000423854	5.11	0.27	0.7085
	GATAD2A	ENST00000358713	9.42	0.02	0.9449
	NCOR1	ENST00000395857	16.2	0.81	**0.0437**
DNA damage response, signal transduction by p53 class mediator	PAXIP1	ENST00000397192	7.02	8.11	**< 0.0001**
	KAT5	ENST00000534650	8.63	6.67	**0.0048**
	E2F7	ENST00000416496	15.1	5.02	**0.0313**
	BRCA1	ENST00000471181	7.78	4.38	0.1271
	NBN	ENST00000396252	6.72	4.00	0.2621
	CNOT9	ENST00000418808	8.62	3.66	0.3269
	BRCA1	ENST00000634433	8.34	3.56	**0.0261**
	MDM2	ENST00000393417	4.68	0.81	0.4343
	RBL2	ENST00000379935	7.77	0.63	0.5348
	GADD45A	ENST00000617962	18.6	0.23	0.4343

*Differentially expressed transcripts with p_adj_ < 0.1 are shown in bold and with p_adj_ < 0.05 are underlined.

The remainder of the 343 oxidation enriched transcripts (291) were not identified as significantly up- or down-regulated. The latter observation suggests that downregulated, oxidation-harboring transcripts may persist longer in the cell relative to those that are replaced by newly synthesized transcripts. It is worth noting that our relative quantitative analysis of transcripts was constrained as following the selected 6-h recovery period post formaldehyde exposures. As such, by focusing on those transcripts enriched in oxidation, we preferentially identify transcripts that are not efficiently turned over or replaced and are therefore more likely to contribute to sustained influences on cellular processes and cell survival; this is justified by the establishment of RNA oxidations as early indicators of cell mortality occurring hours to days after exposure^31^.

## Discussion

In this study, we demonstrate oxidation enrichment of 343 RNA transcripts in cultures exposed to 1 ppm formaldehyde exposure relative to clean air conditions. Although RNA oxidation altered expression levels of many transcripts, this was particularly pronounced on transcripts involved in chromatin modification and DNA-damage response. Given that exposure to 1 ppm formaldehyde caused negligible nucleolar and cytosolic alterations, minimal cytoskeletal defects, and minimal cytotoxicity in BEAS-2B cells, we considered the observed alterations in transcription expression patterns and networks indicative of early cellular responses rather than consequences of apoptosis and necrosis. The ability of formaldehyde to induce specific 8-oxoG modification of RNA molecules and to subsequently compromise the stability of these modified transcripts and their associated pathways represents a potentially novel mechanism for the detrimental and carcinogenic effects of formaldehyde.

Formaldehyde is an environmental and occupational carcinogen broadly used in industrial and consumer products; therefore, it can be found in several domestic products and construction materials^1^. Due to its abundant utilization, formaldehyde is a common indoor pollutant. On average, children and adults spend 90% of the time in indoor environments^84^, suggesting regular exposures to formaldehyde in daily life. Indoor concentrations of formaldehyde typically vary from 5 to 220 µg/m^3^ (4.1 ppb–0.18 ppm)^85^, although these concentrations are mainly reported in industrialized countries with more stringent environmental regulations. Higher levels of formaldehyde are expected in locations with more permissible laws or in places where exposures go unnoticed. For example, mean levels of 670 µg/m^3^ (0.54 ppm) were detected in households using wood for cooking in India^86^. Moreover, levels exceeding 2 ppm have been detected in hair salons during heavy use of hair products containing formaldehyde^87^ (e.g. hair-smoothing products disproportionally used by different demographics).

Given that formaldehyde exposure mainly occurs by inhalation, we used human bronchial epithelial BEAS-2B cells exposed at the air–liquid interface as a model for this study. We found minimal morphological alterations and LDH activity in the media following 1 ppm formaldehyde treatment for 2 h (2 L/min). It is worth noting that similar exposure conditions used in other reports have shown varied responses depending on the cell type, dose, and exposure technique. For example, human alveolar epithelia cell line A549 (a cancerous line) exposed to 1 ppm formaldehyde for 4 h at the ALI (1.0 L/min) rendered a substantial 6.7-fold increase in LDH activity as compared to control samples^44^. Likewise, human tracheal fibroblast Hs 680.Tr cells treated with media containing 100 µM (3 ppm) formaldehyde for 4 h caused ~ 50% reduced viability (determined by MTT colorimetric assay)^88^. Conversely, studies in A549 cells detect a negligible reduction in viability after 0.5 ppm formaldehyde exposure for 72 h using the ALI, and in BEAS-2B cells show over 90% viability over a period of 6 h with exposure concentrations up to 15 ppm^42,89^. The latter observation, also based on BEAS-2B cells, is most consistent with our results, albeit using much higher levels of formaldehyde exposures.

Growing evidence suggests that RNA oxidation might be involved in the pathogenesis of many neurodegenerative diseases (e.g. Alzheimer’s and Parkinson’s disease) given that substantial levels of 8-oxoG-modified RNAs had been detected at early stages of degeneration of vulnerable neurons^90^. Although most research on RNA oxidation has focused on brain disorders, oxidative stress—which is a primary source of RNA oxidation—has been characterized in a broad spectrum of health conditions^91^. Therefore, the association of RNA oxidation in other diseases such as cancers remains to be explored. Environmental factors such as solar UVB radiations and cigarette smoke that have been recognized as risk factors of cancers^92^ can oxidatively damage RNA in the form of 8-oxoG^93,94^. Importantly, observations in the literature argue that due the ability of 8-oxoG to base pair with adenosine, mRNA oxidation can introduce amino acid point mutations^95–97^—the most typical genetic trace in tumors^98^. Moreover, supporting the potential connection of cancer and RNA oxidation, increased free 8-oxoG excretion has been detected in urine of non-small-cell lung cancer patients^99^.

RNA oxidation has been previously profiled using 8-oxoG RIP-seq to identify 8-oxoG accumulation in the cholesterol synthesis transcript FDFT1 in BEAS-2B cells exposed to air pollution mixtures, leading to changes within the cholesterol pathway that result in morphological alterations associated with lung inflammation and fibrosis^39^. Moreover, 8-oxoG profiling in micro RNAs (miRNAs) with 8-oxoG immunoprecipitations coupled to microarrays revealed that oxidized miR-184 erroneously targets transcripts encoding the anti-apoptotic proteins Bcl-xL and Bcl-w for degradation, subsequently inducing initiation of apoptosis in rat heart H9c2 cells during myocardial hypoxia^81^. In this current study, we use 8-oxoG RIP-seq to demonstrate that formaldehyde exposure heavily induces oxidation of transcripts that heavily cluster in the DNA-damage response and signal transduction by p53 class mediator pathways. Moreover, given the impact of 8-oxoG modification on stability of RNA transcripts, several of these transcripts are significantly downregulated. Tumor protein p53 functions as a sequence-specific transcription factor, activated in response to DNA damage generated by diverse environmental stresses such as ionizing radiation, UV light, and methyl methanesulfonate^100^. It promotes genomic stability by regulating many genes responsible in part of G1 phase cell cycle progress^101,102^ and, consequently, p53 prevents tumor progression. Previous works have elucidated that formaldehyde causes point mutations of the p53 gene in nasal squamous cell carcinomas in rats (induced with > 6 ppm formaldehyde), which provoke identical mutations observed in human cancers^103^. Furthermore, formaldehyde exposures experienced at sufficiently high concentrations can compromise genetic stability by DNA fragmentation^43^ and formation of DNA adducts such as N(2)-hydroxymethyl-dG^16^. One of the most functionally connected oxidized transcripts in biological networks found in our Cytoscape network analysis is Breast cancer type 1 susceptibility protein (BRCA1), which plays a central role in DNA repair by facilitating DNA double-strand break repair^104^. BRCA1 mutations confer a high risk of carcinogenesis linked to loss of the ability for cells to maintain genetic integrity^105^. Our indication that formaldehyde induces oxidation of transcripts responsible for DNA-damage responses evidences the early impact of formaldehyde in precluding cells from removing DNA lesions. In fact, loss of p53 signaling and activity is a major driver of cancer because in its absence cells can no longer adequately sustain genome integrity. Taken together, this study presents initial investigations into the role of RNA oxidation in formaldehyde-induced carcinogenesis.

Disruption of chromatin regulation and assembly can contribute to the development of cancer because it induces defects in the DNA repair machinery, essential for genome stability^106^. Mutations in genes involved in chromatin organization and regulation have been identified in over 50% of cancers^107^. Our findings of 21 transcripts in the chromatin modifying enzymes pathway that are extensively oxidized during formaldehyde exposure (13 of these are significantly downregulated) aligns with previous studies that argue the negative effect of formaldehyde in chromatin function. Specifically, formaldehyde exposure dramatically affects acetylation of N-terminal tails of histones^89^. Histone acetyltransferase KAT5, the catalytic subunit of the NuA4 histone acetyltransferase complex, was found among the oxidation enriched transcripts following formaldehyde exposure. This protein complex controls transcriptional activation of several oncogenes (e.g. MYC) by acetylation of histones H4 and H2A^108^. Supporting the connection of formaldehyde-driven deregulation of acetylation of histones and RNA oxidation, we additionally found 8-oxoG accumulation in two transcripts that regulate histones deacetylation, NAD-dependent protein deacetylase sirtuin-1 (SIRT1) and Histone deacetylase 8 (HDAC8). Importantly, SIRT1 deacetylates histone H4 lysine 16 (H4-K16Ac) and histone H3 lysine 9 (H3-K9Ac) and previous studies have linked its upregulation with cellular protection to formaldehyde exposure^109^. HDAC8 deacetylates core histone proteins (e.g., H2A, H2B, H3, and H4) as well as non-histone proteins (e.g., p53)^110^; an activity that is downregulated in several cancers^111^. Besides alteration in acetylation patterns, formaldehyde exposure can also affect inhibition of chromatin assembly^89^. Consistent with this observation, we found that Histone H2A type 1-C (HIST1H2AC), a core component of the DNA packing complex nucleosome, is highly susceptible to oxidation following formaldehyde exposure. Histone-lysine N-methyltransferase SET2D and Histone-lysine N-methyltransferase EHMT2 were also found heavily oxidized by formaldehyde exposure. Notably, SET2D is the main enzyme tri-methylating histone H3 lysine 36 (H3K36me3), a modification involved in DNA repair signaling through homologous recombination and nonhomologous end-joining^112^. The susceptibility to 8-oxoG modification by formaldehyde exposure in transcripts involved in chromatin regulation is likely to reduce genome stability, reflecting RNA oxidation as a novel mechanism implicated in the carcinogenic effects of formaldehyde in cells.

Using 8-oxoG profiling, we also identified many transcripts associated with cancer pathways, including non-small-cell lung cancer, acute myeloid leukemia, and prostate cancer (Table 2). Among these oxidized transcripts, Signal transducer and activator of transcription 3 (STAT3) and E3 ubiquitin-protein ligase MDM2 are important regulators of carcinogenesis^113,114^. Although neither of these transcripts were significantly regulated, the detection of 8-oxoG modifications in cancer-related transcripts indicates that together with more general processes in carcinogenesis such as DNA damage response and chromatin regulation, oxidation of guanine occurs disproportionately on transcripts associated with specific pathways in different types of cancers. Overall, we provide a first approximation to understanding post-transcriptional effects of formaldehyde in key pathways and processes that are linked to cancer development. Given that these observations are identified in normal cells without exhibiting cell death phenotypes (i.e. nuclear shrinkage and fragmentation or increased LDH activity), we hypothesize that oxidative damage of specific RNA transcripts following sublethal formaldehyde exposure could represent an early process occurring in carcinogenesis, analogous to the events of significant increase of 8-oxoG-modified transcripts at early stages of neurodegenerative diseases^31,115^. The validation of the formation of 8-oxoG in specific transcripts reported in this study requires investigation in further work, using the workflows described previously^28,81^. Moreover, it is important to confirm whether similar responses to formaldehyde exposure can be observed in other models of respiratory epithelial cells. Nevertheless, our studies provide initial insights into a new mechanism underlying formaldehyde carcinogenesis and toxicity in cells.

**Table 2:** Fold-change in 8-oxoG enrichment in transcripts involved in cancer pathways after formaldehyde exposure.

Cancer type	HGNC symbol	Ensembl transcript ID	8-oxoG enrichment log_2_(FC)
Acute myeloid leukemia	STAT3	ENST00000389272	13.4
	IKBKB	ENST00000520810	9.59
	RELA	ENST00000612991	8.53
	ZBTB16	ENST00000392996	7.73
	DUSP6	ENST00000547140	6.12
Non-small-cell lung cancer	FN1	ENST00000336916	11.5
	IKBKB	ENST00000520810	9.59
	FN1	ENST00000359671	8.65
	RELA	ENST00000612991	8.53
	GADD45A	ENST00000617962	7.77
	ITGA6	ENST00000442250	7.45
	ITGA2	ENST00000503810	5.01
Prostate cancer	CTNNB1	ENST00000643052	10.1
	IKBKB	ENST00000520810	9.59
	RELA	ENST00000612991	8.53
	MDM2	ENST00000393417	8.34
	ZEB1	ENST00000542879	7.76
	PLAT	ENST00000429089	6.52

## Conclusions

The results presented herein support the growing body of evidence that specific transcripts are more prone to RNA oxidation than others and that transcript oxidation is not a random event. Our study identified significant enrichment of RNA oxidation on transcripts associated with specific pathways, including those previously indicated in the cellular response to formaldehyde exposure, suggestive of a functional role. At biologically relevant conditions of the formaldehyde exposure tested here (1 ppm for 2 h) followed by 6 h of recovery, we identified enrichment of oxidized transcripts associated with pathways including chromatin modification and DNA-damage response by p53, demonstrating a sustained presence of these oxidized transcripts. Considering that previous research has demonstrated translational mutations resulting from RNA oxidation, altered proteins produced by these transcripts could be indicative of early pathway dysregulation and disease onset. Our work strengthens previous studies pointing to RNA transcripts as candidates for biomarkers of oxidative stress or early onset of disease^39,50,116^ given their prolonged enrichment of oxidation. Based on the functional relevance of oxidation enriched transcripts on interacting cellular processes, we propose RNA oxidation as an additional driver of cell physiology, health, and disease that remains undetected by standard transcriptomics approaches. As technology progresses in this field, we anticipate that the identification of 8-oxoG modifications at nucleotide resolution will allow more insight to the biological implications of oxidations on specific transcripts. In the context of environmental toxins, utilization of molecular reporters, like oxidized RNAs, that can report early alterations to functional cellular pathways will be key in protecting consumers, employees, and health care workers from the deleterious effects of harmful chemicals (like formaldehyde) ubiquitously present in manufactured goods.

## Supplementary information

Supplementary Information.

Supplementary Information.
