# Supplementary material for: RNA oxidation in chromatin modification and DNA-damage response following exposure to formaldehyde
Source: Sci Rep. 2020 Oct 6;10:16545. doi: 10.1038/s41598-020-73376-7 (PMC7538935; doi:10.1038/s41598-020-73376-7)
Supplement: Supplementary file 2 — Supplementary Information. [file 41598_2020_73376_MOESM2_ESM.docx]

Supplementary Information for

RNA oxidation in chromatin modification and DNA-damage response following exposure to formaldehyde

Juan C. Gonzalez^‡^, Mark W. Sherman^‡^, Dongyu S. Wang, Jamie C. L. Chuvalo‐Abraham, Lea Hildebrandt Ruiz, and Lydia M. Contreras

^‡^Authors contributed equally to this work

Lydia M. Contreras

Email: lcontrer@che.utexas.edu

**This PDF file includes:**

Supplementary Methods

Figure S1 – S3

**Other supplementary materials for this manuscript include the following:**

Dataset 1: S1 – S9

## Supplemental Methods

### Formaldehyde Exposure

**Table M1.** The amount of paraformaldehyde powder and resulting gas concentration for each exposure.

| Date | Powder weighed (mg+0.1) | Gas concentration (ppb) |
| --- | --- | --- |
| 2018/07/12 | 12.8 | 1040 |
| 2018/07/14 | 13.2 | 1070 |
| 2018/07/16 | 12.6 | 1030 |

The conversion of the paraformaldehyde powder mass to gas-phase formaldehyde concentration (Table M1) was performed assuming complete powder volatilization and a chamber volume of 10 m^3^ at standard conditions (1 atm and 298 K), according to Equation 1,

$C=M\times{10}^{3}\times\left( 0.0409*MW \right)^{-1}\times\left( 10 \right)^{-1}$ (1)

where *C* is the gas-phase formaldehyde concentration in ppb, *M* is the mass of paraformaldehyde powder weighed in mg, and *MW* is the molecular weight of formaldehyde (30.031 g mol^-1^).

A high-resolution time-of-flight chemical ionization mass spectrometer (CIMS, Aerodyne, Billerica, MA) was used to monitor the molecular composition of gas-phase compounds using (H_2_O)_n_H_3_O^+^ as the chemical ionization reagent in the positive ion detection mode^1^. Similar to a proton transfer mass spectrometer (PTR-MS), the (H_2_O)_n_H_3_O^+^ was used to detect unoxidized (e.g. sesquiterpenes and monoterpenes) to lightly oxidized volatile organic compounds (e.g. alcohols, ketones, and aldehydes) whose proton affinity is higher than that of the hydronium (and of hydronium-water clusters). Whereas the hydronium ion is the dominant chemical ionization reagent in the PTR-MS, the number of water clusters, *n* ranged between 0 to 2 for (H_2_O)_n_H_3_O^+^ CIMS, with (H_2_O)H_3_O^+^ being the dominant chemical ionization reagent ion. As the proton affinity increases from hydronium to hydronium-water cluster(s), the (H_2_O)_n_H_3_O^+^ is less sensitive than the PTR-MS and exhibits more complex clustering and fragmentation patterns in the mass spectra. Both the proton transfer (Reaction 2) and the ligand exchange (Reaction 3) ion products corresponding to gas-phase formaldehyde were observed,

$CH{}_{2}O+H_{3}O^{+}\to\left( CH{}_{2}O \right)H^{+}+H{}_{2}O$ (2)

$CH{}_{2}O+(H_{2}O)H_{3}O^{+}\to\left( CH{}_{2}O \right)H_{3}O^{+}+H{}_{2}O$ (3)

Additional organic compounds such as CH_3_OH (methanol), C_2_H_5_OH (ethanol), C_2_H_4_O (acetaldehyde), were also observed during formaldehyde injection and seen to increase in proportion to the amount of formaldehyde added, which we have attributed to impurities present in the paraformaldehyde powder itself (Figure M1).


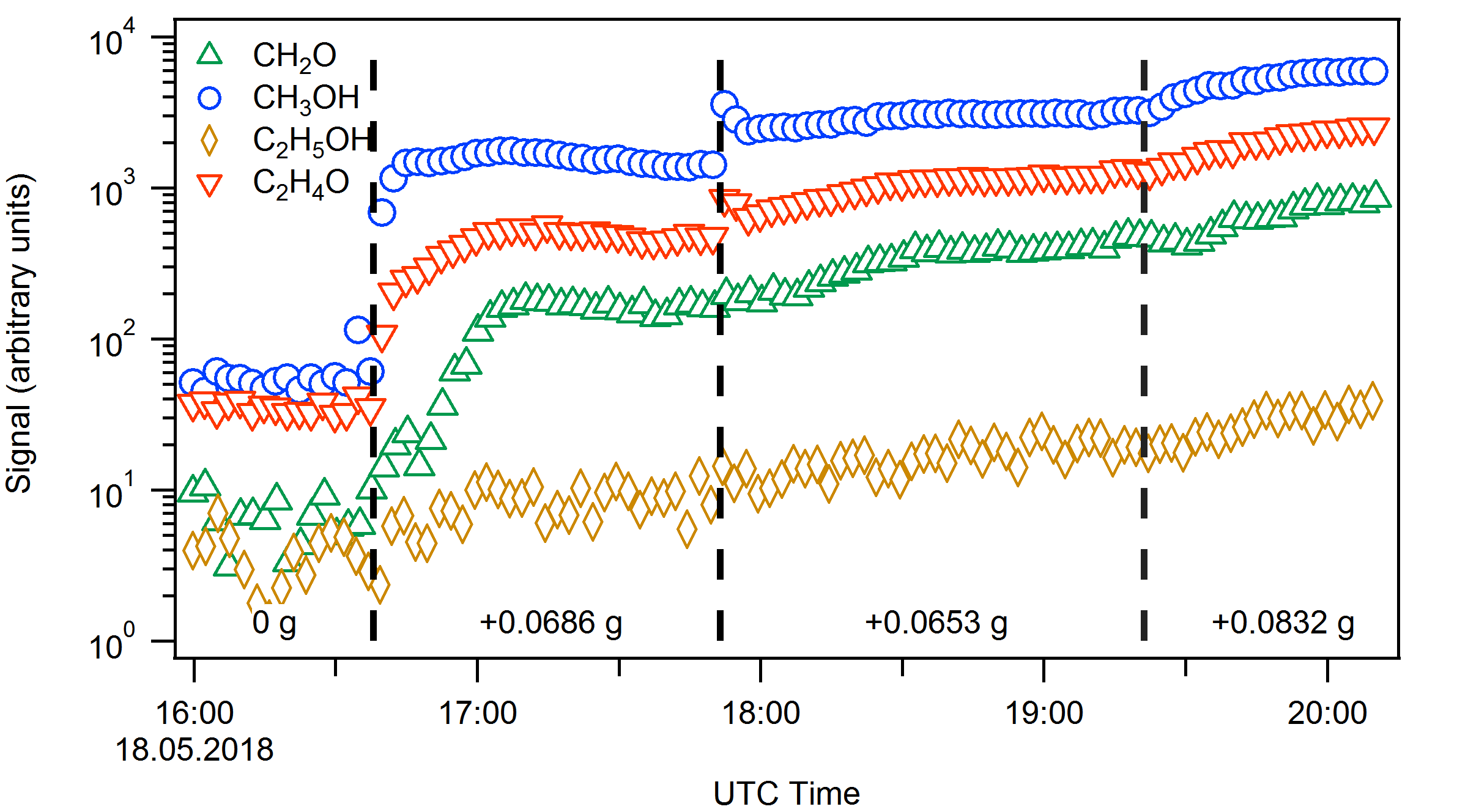


**Figure M1.** Time-series of select gas-phase compounds observed by the (H_2_O)_n_H_3_O^+^ CIMS during formaldehyde injection. The beginning of each injection event is indicated by vertical dash lines. Amounts of paraformaldehyde power added are indicated in the annotations. Compounds consistent with methanol (CH_3_OH) [Blue circles], acetaldehyde (C_2_H_4_O) [red triangles], and ethanol (C_2_H_5_OH) [purple diamonds] were observed in addition to formaldehyde (CH_2_O) [green triangles]. Ion intensities were normalized against reagent ion signal, which was assumed to be 10^6^ cps.

The CIMS was also operated in the negative ion mode using (H_2_O)_n_I^-^ as the chemical ionization reagent ion to track organic acid impurities^2^. The number of water clusters, *n* ranges between 0 to 1 for (H_2_O)_n_I^-^ CIMS, with I^-^ being the dominant chemical ionization reagent ion. Although deprotonated product ions were observed in the mass spectra, only the iodide-adduct ions (Reaction 4) were used to monitor change concentration of a gas-phase species, X, where

$X+I^{-}\to XI^{-}$ (4)

No correlation was observed between formaldehyde injection and formic acid concentration. Compounds consistent with glycolic acid (C_2_H_4_O_3_) and lactic acid (C_3_H_6_O_3_) were observed during formaldehyde injection on 2018-07-14 but not observed during exposures on 2018-07-12 or 2018-07-16 (Figure M2). The source of these organic acid contaminations may be from skin contact or organic residues on the Swagelok pieces. The glycolic acid and lactic acid contaminations decreased to near-background levels shortly after (within 15 minutes) the end of formaldehyde injection (Figure M2). CH_2_O_2_ (blue), C_2_H_4_O_3_ (red), and C_3_H_6_O_3_ (green) do not show consistent patterns across the three exposure dates, suggesting that contamination is not systematic. During the 2018-07-14 experiment, concentrations consistent with glycolic acid (C_2_H_4_O_3_) and lactic acid (C_3_H_6_O_3_) increased during formaldehyde injection but decreased back to near-background levels over the course of 30 minutes. Increase in formic acid (CH_2_O_2_) concentrations later on, which was not observed during other exposure experiments, may be due to leaks in the environmental chamber wall.


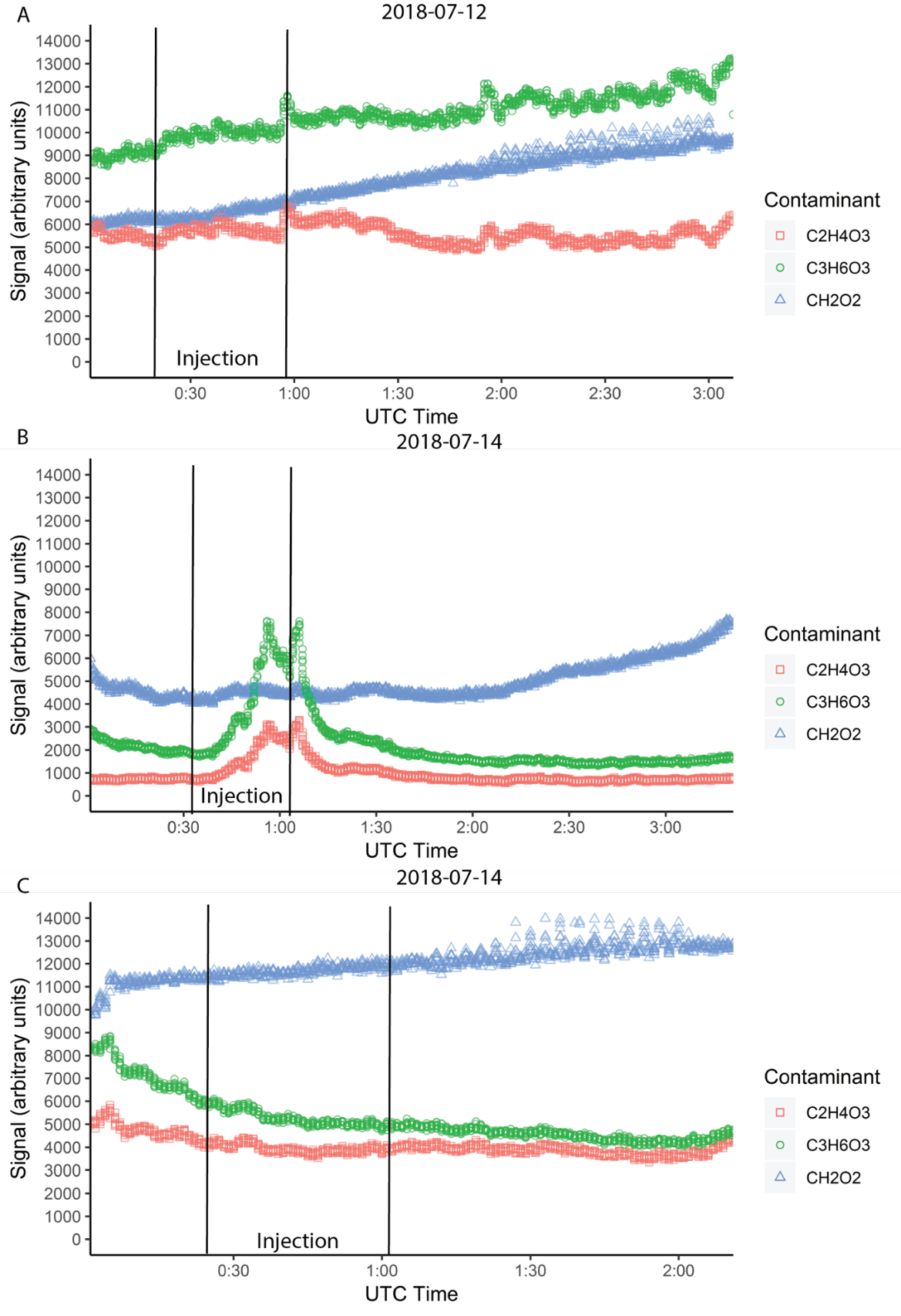


**Figure M2.** Time-series of gas-phase organic acid contaminations measured during each of the exposures detected through mass spectrometry. (A) 2018-07-12, (B) 2018-07-14, and (C) 2018-07-16. Contaminants are plotted over time CH_2_O_2_ [blue triangles], C_2_H_4_O_3_ [red squares], and C_3_H_6_O_3_ [green circles]

### Data Analysis

Raw sequencing data was acquired from the DNA sequencing core facilities at the University of Texas at Austin. Sequences were visually assessed by FastQC (<https://www.bioinformatics.babraham.ac.uk/index.html)> for run quality. Runs were then processed with cutadapt to remove primer and adaptor sequences^3^.

cutadapt -a GATCGGAAGAGCACACGTCTGAACTCCAGTCACACAGTGATCTCGTATGCCGTCTTCTGCTTG -A GATCGGAAGAGCACACGTCTGAACTCCAGTCACACAGTGATCTCGTATGCCGTCTTCTGCTTG -q 30 -o in-CA-R1_S1_L001_R1.trimmed.fastq -p in-CA-R1_S1_L001_R2.trimmed.fastq in-CA-R1_S1_L001_R1_001.fastq.gz in-CA-R1_S1_L001_R2_001.fastq.gz

FastQC was performed again to ensure read quality after trimming and data was assessed for any anomalies or repetitive sequences. Trimmed reads were then aligned by STAR aligner to ENSEBL GRCh38.p12 primary genome assembly (<ftp://ftp.ensembl.org/pub/release-94/fasta/homo_sapiens/dna/Homo_sapiens.GRCh38.dna_sm.primary_assembly.fa.gz)> with the corresponding annotations (<ftp://ftp.ensembl.org/pub/release-94/gtf/homo_sapiens/Homo_sapiens.GRCh38.94.gtf.gz)>^4^.

Genome construction:

STAR --runMode genomeGenerate --genomeDir . --genomeFastaFiles Homo_sapiens.GRCh38.dna_sm.primary_assembly.fa --sjdbGTFfile Homo_sapiens.GRCh38.94.gtf --sjdbOverhang 74 --genomeChrBinNbits 14

First pass of STAR mapping:

/STAR \

--runThreadN 24 \

--genomeDir \

/HumanGenome/GRCh38.94 \

--readFilesIn in-FA-R3_S4_R1.trimmed.fastq in-FA-R3_S4_R2.trimmed.fastq \

--genomeLoad NoSharedMemory \

--outFilterIntronMotifs None \

--outSAMattrIHstart 0 \

--outSAMstrandField intronMotif \

--outFilterType BySJout \

--outFilterMultimapNmax 20 \

--alignSJoverhangMin 8 \

--alignSJDBoverhangMin 1 \

--outFilterMismatchNmax 999 \

--outFilterMismatchNoverLmax 0.04 \

--alignIntronMin 20 \

--alignIntronMax 1000000 \

--alignMatesGapMax 1000000 \

--outSAMtype BAM SortedByCoordinate \

--outFileNamePrefix "./in-FA-R3_S4."

Splice junctions were then compiled for 2nd pass mapping as recommended by the STAR manual.

Second pass mapping:

/STAR \

--runThreadN 24 \

--genomeDir GRCh38.94 \

--readFilesIn IP-FA-R1_S7_R1.trimmed.fastq IP-FA-R1_S7_R2.trimmed.fastq \

--outReadsUnmapped Fastx \

--outSAMtype BAM SortedByCoordinate \

--quantMode TranscriptomeSAM GeneCounts \

--outBAMcompression 10 \

--quantTranscriptomeBAMcompression 10 \

--outFilterIntronMotifs None \

--outSAMattrIHstart 0 \

--outFilterType Normal \

--outFilterMultimapNmax 20 \

--alignSJoverhangMin 8 \

--alignSJDBoverhangMin 1 \

--outFilterMismatchNmax 999 \

--outFilterMismatchNoverLmax 0.04 \

--alignIntronMin 20 \

--alignIntronMax 1000000 \

--alignMatesGapMax 1000000 \

--sjdbFileChrStartEnd ./in-CA-R1_S1.SJ.out.tab ./in-CA-R3_S2.SJ.out.tab ./in-FA-R1_S3.SJ.out.tab ./in-FA-R3_S4.SJ.out.tab ./IP-CA-R1_S5.SJ.out.tab ./IP-CA-R3_S6_R1.SJ.out.tab ./IP-FA-R1_S7.SJ.out.tab ./IP-FA-R3_S8.SJ.out.tab \

--outFileNamePrefix "./IP-FA-R1."

Read alignments were visually inspected by Inter Genome Viewer and RSEM (<https://deweylab.github.io/RSEM/)> reference file was the prepared by RSEM to use with RSEM to count reads associated with splice variants^5,6^.

RSEM reference generation:

/rsem-prepare-reference \

--gtf \

/Homo_sapiens.GRCh38.94.gtf \

/Homo_sapiens.GRCh38.dna_sm.primary_assembly.fa \

/GRCh38.rsem.ref.genome

RSEM was used to estimate the number of reads assigned to each splice variant.

/rsem-calculate-expression \

--paired-end \

-p 24 \

--bam \

--output-genome-bam \

/in-CA-R1_S1_R2.Aligned.toTranscriptome.out.bam \

/GRCh38.rsem.ref.genome \

/in-CA-R1_S1


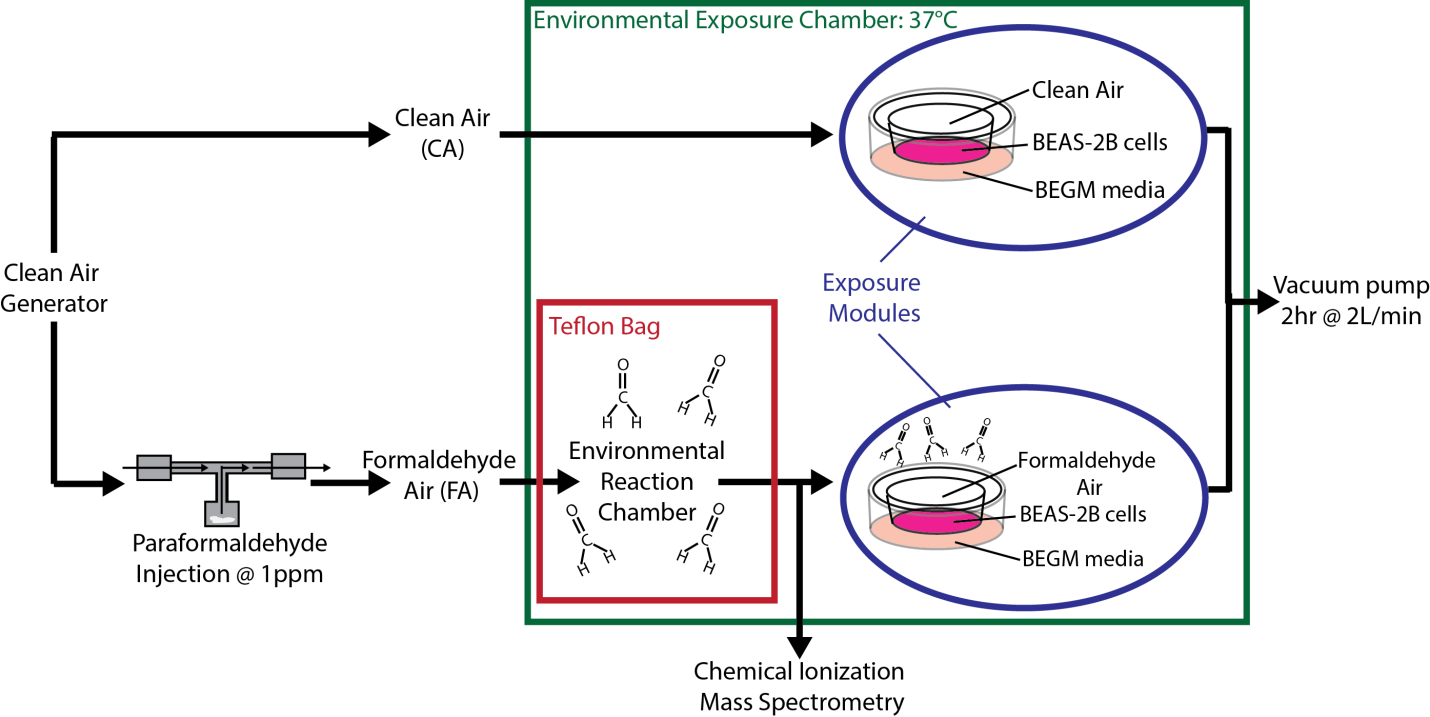


**Figure S1.** Formaldehyde injection and exposure system. Clean air was generated with an high air purity generator (AADCO instruments) and channeled either into the clean air exposure chamber or injected with formaldehyde generated by thermal decomposition of paraformaldehyde powder into the environmental reaction chamber, then pulled into the formaldehyde exposure chamber at 2 L/min. BEAS-2B cell cultures were either exposed to the formaldehyde-air mix or clean air for two hours at 37˚C and recovered for 6 hours in fresh media at 37˚C before processing.


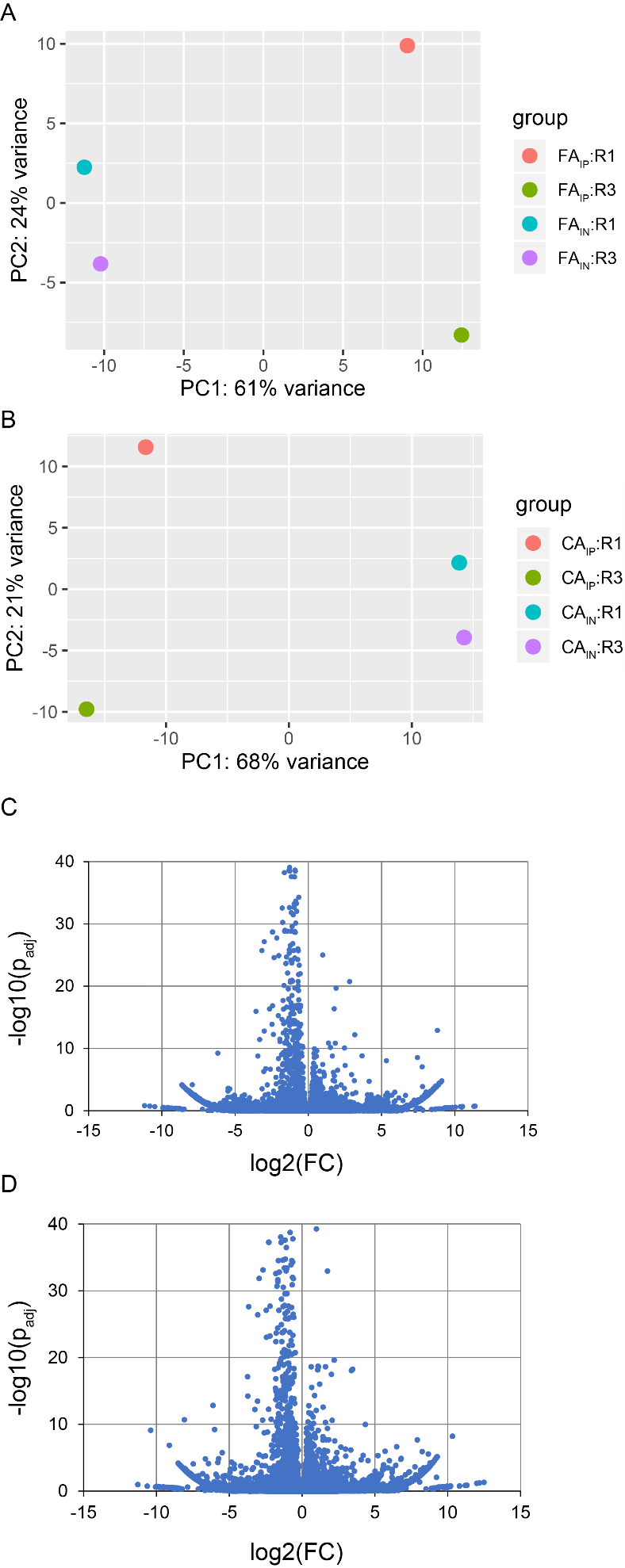


**Figure S2.** Statistical analysis of 8-oxoG RIP-seq data from BEAS-2B cells exposed to formaldehyde (FA) relative to clean air (CA) controls using DESEQ2 show major drivers of variance. Principal component analysis (PCA) of full transcriptomics for formaldehyde (FA) exposure samples FA_IP_ vs FA_IN_ (A) and for clean air (CA) controls CA_IP_ vs CA_IN_ (B). IN and IP refers to input RNA pool previous and post-immunoprecipitation, respectively. PCA 1 and PC2 represent the % of explained variances. Biological replicas are shown in the legend box (i.e. R1 and R3) and spatial proximity among replicas indicate similar patterns of expression. Volcano plot of full transcriptomics for formaldehyde (FA) exposure samples for FA_IP_ vs FA_IN_ (C) and for clean air (CA) controls CA_IP_ vs CA_IN_ (D). Full transcriptomics data after DESEQ2 analysis used to create the volcano plots is available in Dataset S7 and S8.


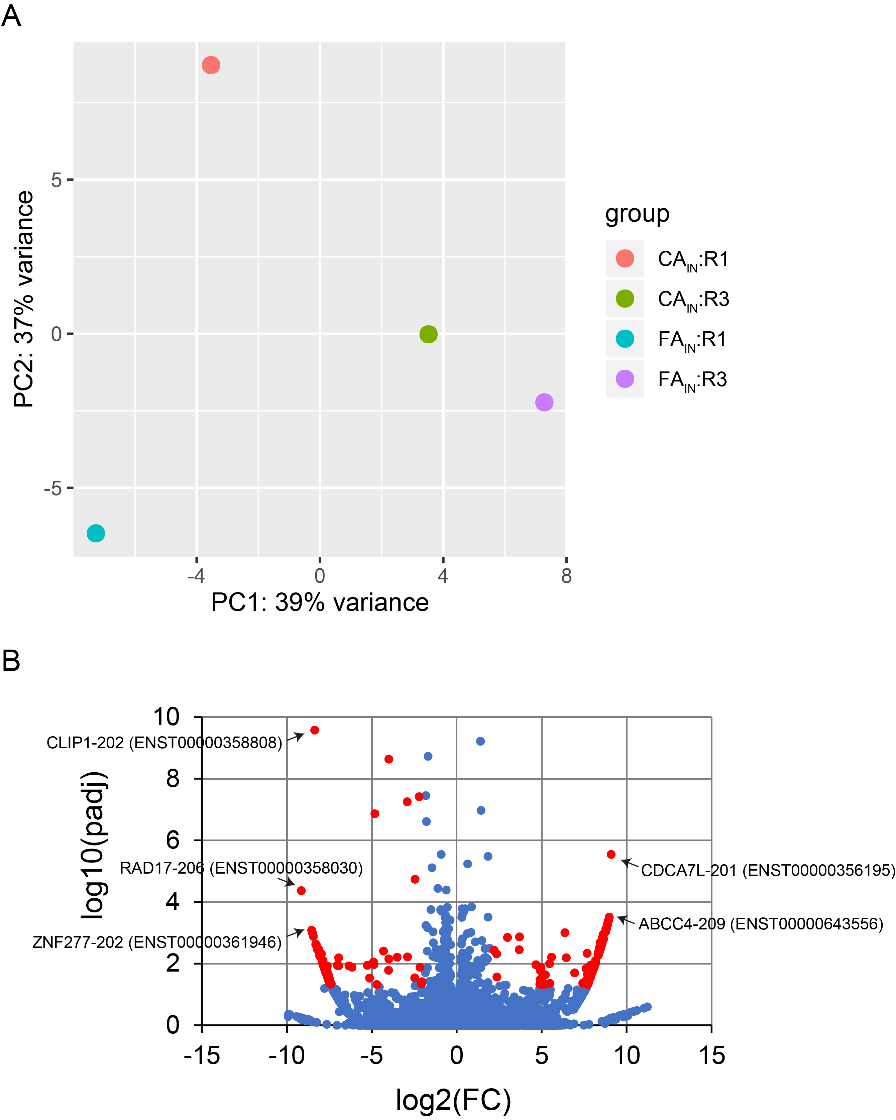


**Figure S3.** Statistical analysis of the RNA-seq data from BEAS-2B cells exposed to formaldehyde (FA) relative to clean air (CA) controls using DESEQ2 show major drivers of variance. (A) Principal component analysis (PCA) of full transcriptomics pre-immunoprecipitation for formaldehyde (FA_IN_) and clean air (CA_IN_). PCA 1 and PC2 represent the % of explained variances. Biological replicas are shown in the legend box (i.e. R1 and R3) and spatial proximity among replicas indicate similar patterns of expression. Volcano plot of full transcriptomics for formaldehyde (FA_IN_) exposure samples and clean air (CA_IN_) controls. Red color indicates differential expressed transcripts (log2(FC)>|2| and p_adj_ < 0.05). Full transcriptomics data after DESEQ2 analysis used to create the volcano plots is available in Dataset S9.

## Supplemental works cited

1. Aljawhary, D., Lee, A. K. Y. & Abbatt, J. P. D. High-resolution chemical ionization mass spectrometry (ToF-CIMS): Application to study SOA composition and processing. *Atmos. Meas. Tech.* **6**, 3211–3224 (2013).

2. Lee, B. H. *et al.* An iodide-adduct high-resolution time-of-flight chemical-ionization mass spectrometer: Application to atmospheric inorganic and organic compounds. *Environ. Sci. Technol.* **48**, 6309–6317 (2014).

3. Martin, M. Cutadapt removes adaptor sequences from high-throughput sequencing reads. *EMBnet.journal* **17**, 10–12 (2011).

4. Dobin, A. *et al.* STAR: Ultrafast universal RNA-seq aligner. *Bioinformatics* **29**, 15–21 (2013).

5. Thorvaldsdóttir, H., Robinson, J. T. & Mesirov, J. P. Integrative Genomics Viewer (IGV): High-performance genomics data visualization and exploration. *Brief. Bioinform.* **14**, 178–192 (2012).

6. Li, B. & Dewey, C. N. RSEM: Accurate transcript quantification from RNA-seq data with or without a reference genome. *BMC Bioinformatics* **12**, 1–16 (2014).
